# Society Notes

**Published:** 1903-02

**Authors:** 


### SOCIETY NOTES.

American Veterinary Medical Association. Local Committee of Arrangements, Ottawa, Canada, September 1 to 4, 1903: Drs. J. G. Rutherford (Chairman), C. H. Higgins, Ottawa; Wm. Jakeman, Halifax; G. Alarie, Comte l'Assomption, Quebec; D. King Smith, Ontario; F. Torrance, Manitoba; J. B. Hart, British Columbia.

The Allegheny Veterinary Medical Association will shortly extend its usefulness and power by changing its name to the Western Pennsylvania Veterinary Medical Association.

